# Bacterial and fungal growth in sputum cultures from 165 COVID-19 pneumonia patients requiring intubation: evidence for antimicrobial resistance development and analysis of risk factors

**DOI:** 10.1186/s12941-021-00472-5

**Published:** 2021-09-25

**Authors:** Hans H. Liu, David Yaron, Amanda Stahl Piraino, Luciano Kapelusznik

**Affiliations:** 1 Division of Infectious Diseases, Department of Medicine, Bryn Mawr Hospital, Main Line Health System, Bryn Mawr, PA USA; 2 Department of Family Medicine, Bryn Mawr Hospital, Main Line Health System, Bryn Mawr, PA USA; 3 Sidney Kimmel Medical College, Thomas Jefferson University, Philadelphia, PA USA; 4 219 Garnet Lane, Bala Cynwyd, PA 19004 USA

**Keywords:** SARS-CoV-2, COVID-19, Pneumonia, Bacterial superinfection, Sputum culture, Antibiotic resistance, Antimicrobial stewardship

## Abstract

**Background:**

Coronavirus SARS-CoV-2 causes COVID-19 illness which can progress to severe pneumonia. Empiric antibacterials are often employed though frequency of bacterial coinfection superinfection is debated and concerns raised about selection of bacterial antimicrobial resistance. We evaluated sputum bacterial and fungal growth from 165 intubated COVID-19 pneumonia patients. Objectives were to determine frequency of culture positivity, risk factors for and outcomes of positive cultures, and timing of antimicrobial resistance development.

**Methods:**

Retrospective reviews were conducted of COVID-19 pneumonia patients requiring intubation admitted to a 1058-bed four community hospital system on the east coast United States, March 1 to May 1, 2020. Length of stay (LOS) was expressed as mean (standard deviation); 95% confidence interval (95% CI) was computed for overall mortality rate using the exact binomial method, and overall mortality was compared across each level of a potential risk factor using a Chi-Square Test of Independence. All tests were two-sided, and significance level was set to 0.05.

**Results:**

Average patient age was 68.7 years and LOS 19.9 days. Eighty-three patients (50.3% of total) originated from home, 10 from group homes (6.1% of total), and 72 from nursing facilities (43.6% of total). Mortality was 62.4%, highest for nursing home residents (80.6%). Findings from 253 sputum cultures overall did not suggest acute bacterial or fungal infection in 73 (45%) of 165 individuals sampled within 24 h of intubation. Cultures ≥ 1 week following intubation did grow potential pathogens in 72 (64.9%) of 111 cases with 70.8% consistent with late pneumonia and 29.2% suggesting colonization. Twelve (10.8% of total) of these late post-intubation cultures revealed worsened antimicrobial resistance predominantly in *Pseudomonas*, *Enterobacter, or Staphylococcus aureus*.

**Conclusions:**

In severe COVID-19 pneumonia, a radiographic ground glass interstitial pattern and lack of purulent sputum prior to/around the time of intubation correlated with no culture growth or recovery of normal oral flora ± yeast. Discontinuation of empiric antibacterials should be considered in these patients aided by other clinical findings, history of prior antimicrobials, laboratory testing, and overall clinical course. Continuing longterm hospitalisation and antibiotics are associated with sputum cultures reflective of hospital-acquired microbes and increasing antimicrobial resistance.

*Trial registration*: Not applicable as this was a retrospective chart review study without interventional arm.

## Background

Coronavirus SARS-CoV-2 causes COVID-19 disease typically presenting with fever, cough, fatigue and dyspnea [1]. Pulmonary symptoms may follow direct viral invasion and later immune-mediated “cytokine storm” [2, 3]. In severe COVID-19, clinical features often are consistent with sepsis and septic shock [4]. Lung pathology reflects viral injury, bacterial superinfection, or immune-mediated endothelitis and microthrombosis [5, 6]. In the United States, COVID-19 infections and associated hospitalisations, intensive care unit (ICU) utilization, and ventilator usage surged in Spring and Fall 2020. Diagnosis and management of individuals with fever and respiratory disease associated with COVID-19 remain challenging and therapeutic approaches continue to evolve [7, 8].

Respiratory viral infections, especially influenza, are associated with bacterial and fungal superinfection [9–11]. This has also been noted in previous coronavirus outbreaks of Severe Acute Respiratory Syndrome (SARS) [12] and Middle Eastern Respiratory Syndrome (MERS) [13]. However, there is relatively little data on prevalence and severity of bacterial and fungal superinfections in COVID-19. Current World Health Organization guidance does not recommend routine antibiotic treatment in COVID-19 though this applies to moderate disease without clinical suspicion of bacterial infection [14]. Severe COVID-19 with pulmonary infiltrates and a septic picture progressing to respiratory failure commonly leads to antimicrobial agents directed at bacterial superinfection [15–18]. Use of immunosuppressants such as tocilizumab and dexamethasone [19, 20] to combat COVID-19 cytokine storm also contributes to empiric antibacterial and antifungal therapy [21]. This in turn raises concerns about development of antimicrobial resistance (AMR) [22–25].

Preliminary data suggested bacterial and fungal superinfection may be less common in COVID-19 pneumonia than in influenza [26, 27]. Initial studies were predominantly from China [28] with a need for larger study numbers, greater geographic distribution, and longer follow up of patients suggested [29, 30]. Recently published microbiologic studies come from China [7], Europe [16, 17, 31–33] and the Americas [34, 35]; most studies have been retrospective and based on a hospitalized population. However, some present data collected during the early stages of COVID-19 infection and more likely reflect community-acquired infection [18]. Others compile cultures from long duration hospital stays and include nosocomial infections but may not clearly differentiate early versus late infections, define hospital-acquired pneumonia (HAP) versus ventilator-associated pneumonia (VAP), or clearly delineate infection from respiratory tract colonization. Accordingly characterization of positive respiratory cultures by time course and differentiation of infection from colonization was desirable. Optimal pneumonia management in COVID-19 patients would reduce prolonged antibiotic courses, restrain development of multidrug-resistant pathogens, and conserve hospital resources [36, 37].

## Methods

### Study setting

The Main Line Health System (MLHS) consists of five hospitals just northwest of Philadelphia, Pennsylvania, in the mid-Atlantic United States. The system’s four acute care teaching hospitals total 1058 beds including 138 ICU beds. MLHS hospitals began seeing COVID-19 in early March 2020. MLHS had 801 total COVID-19 discharges by May 1, 2020; individual hospitals saw 121–323 discharges.

### Ethical approval and data collection

After Institutional Review Board approval, we compiled a list of patients with positive nasopharyngeal polymerase chain reaction (PCR) assays for SARS-CoV-2 virus who were intubated and admitted to ICU between March 1 and May 1, 2020, inclusive. Cases were reviewed individually online. Analysis used FileMaker^(R)^ database software on secure computers. Of 188 total patients, 23 were excluded due to lack of positive SARS-CoV-2 PCR within study dates (3 patients), no radiographic evidence of pneumonia during admission (3), death within 48 h of admission (13), palliative care chosen on admission (1), and ICU admission due to non-COVID-19-related critical illness (3). Cases meeting study criteria were reviewed from admission until discharge or death; one patient was still hospitalised after 90 days when study follow up ended.

### Prior and concurrent medications including antibiotics

Of 165 patients meeting study criteria, 34 (20.6%) had received antibiotics prior to admission. Regimens encountered more than once were azithromycin (10), amoxicillin/clavulanate (3), cephalexin/cefuroxime (3), levofloxacin (3), ceftriaxone (3), and TMP-SMX (2). Admission protocol at the time called for 5 days hydroxychloroquine (HCQ) and azithromycin as potential treatment for COVID-19; almost all patients received HCQ and 78 (47.3%) of patients received azithromycin. Exceptions had allergic or QTc prolongation contraindications to these drugs and azithromycin use decreased substantially by early April 2020 based on lack of demonstrated efficacy.

Following admission, of the 165 patients reviewed, 123 required vasopressors, 54 were given tocilizumab, and 7 received extracorporeal membrane oxygenation (ECMO). Additionally, at intubation 144 (87.3%) were started on empiric antimicrobials directed at potential concurrent bacterial pneumonia; most commonly used were ceftriaxone in 59 (= 41% of regimens), a different 3rd generation cephalosporin in 8 (5.6%), vancomycin + 3rd generation cephalosporin ± metronidazole in 25 (17.4%), vancomycin + piperacillin/tazobactam in 12 (8.3%), piperacillin/tazobactam in 2 (1.4%), and fluoroquinolone in 2 (1.4%).

### Definitions and data analysis

Each sputum culture obtained or ordered was evaluated; cultures within 48 h of each other with identical results were considered a single culture. Results were categorized as no sputum obtained, ordered/not done, without growth, contaminated, or based on organism(s) recovered. Clinical significance was defined:

*Pneumonia on admission without intubation*: radiographic evidence of pneumonia within 72 h of admission but ≥ 4 days prior to intubation.

*Pneumonia within 24 h of intubation*: radiographic evidence of pneumonia during the 24 h before or after intubation.

*Late pneumonia*: radiographic evidence of worsening pneumonia occurring more than 7 days since any prior pneumonia diagnosis and treatment.

*Colonization*: growth of organism(s) other than normal oral flora ± yeast without radiographic evidence of a change in infiltrate appearance; no antibiotic ordered in response.

*Antibiotic course*: antimicrobial therapy ≥ 5 days directed against documented or suspected respiratory pathogen(s); completed or interrupted by patient death/change to palliative care.

Episodes of pneumonia or colonization had to be separated from other episodes by at least 7 days. Determination of infection versus colonization was based on review of contemporaneous notes of attending infectious diseases consultants, pulmonary critical care specialists, and/or internist/hospitalists; in addition, hospital antibiotic orders helped distinguish diagnoses of infection from colonization, and discharge summaries were used to confirm pneumonia diagnoses. Radiographic evidence of pneumonia were based on a radiologist’s CXR or CT scan official report citing new evidence of pneumonia or changes suggestive of worsening pneumonia.

*Development of antimicrobial resistance (AMR)*: Repetitive sputum isolation during a patient's hospitalisation of the same microorganism which had acquired significantly increased antimicrobial resistance determinates compared with original isolate.

Sputum cultures were judged to be less suggestive of pyogenic bacterial infection if no sputum was obtained, culture was without growth, or only normal oral flora ± yeast was isolated; potential for prior antimicrobial therapy suppressing potential respiratory pathogens was a concern in certain subgroups.

### Statistical methods

Length of stay (LOS) is described as mean (standard deviation) and categorical variables are described as frequency or percentage. A 95% confidence interval (95% CI) was computed for overall mortality rate using the exact binomial method, and overall mortality was compared across each level of a potential risk factor using a Chi-Square Test of Independence. All tests were two-sided, and significance level was set to 0.05. Data was analyzed in Stata/MP 15.1 (StataCorp LP., Texas, USA).

## Results

### Demographics of study population and clinical outcomes

Table 1 shows characteristics and clinical outcomes for 165 patients studied. Overall mortality was 62.4% (95% CI: 54.6, 69.8%). Men constituted 55.8% of patients; mortality was 57.6% for men compared with 68.5% for women (p = 0.152). Mortality increased with increasing age and patients admitted from home had a mortality of 45.8% compared with 70% in those coming from group homes (p = 0.148) and 80.6% from nursing homes (p ≤ 0.001). History of hypertension, obesity (body mass index > 30), smoking-related issues, and diabetes mellitus were common but did not have statistically significant associations with mortality.

**Table 1:** Demographics and risk factors bearing on outcomes for 165 COVID-19 patients

Category/risk factor	# Patient (% of total)	# Died (% of total)	p-value	LOS (survivors) days, Mean
All patients	165(100%)	103 (62.4%)		28.5
Sex			0.152	
Male	92 (55.8%)	53 (57.6%)		27.5
Female	73 (44.2%)	50 (68.5%)		30.1
Age			< 0.001	
< 65 years	61 (37%)	26 (42.6%)		25.9
65 to 80 years	71 (43%)	49 (69%)		33.1
> 80 years	33 (20%)	28 (84.9%)		25.8
Race			0.094	
White	71 (43%)	50 (70.4%)		27.2
Black	86 (52.1%)	50 (58.1%)		27.6
Asian, hispanic, other	8 (4.9%)	3 (37.5%)		40
Residence			< 0.001	
Home	83 (50.3%)	38 (45.8%)		29.2
Group home	10 (6.1%)	7 (70%)		23.7
Nursing facility	72 (43.6%)	58 (80.6%)		27.2
				LOS until death (days)
Age vs. LOS until death				
< 65 years	61 (37%)	26 (42.6%)		16.7
65–80 years	71 (43%)	49 (69%)		14.2
> 80 years	33 (20%)	28 (84.8%)		13.8
Residence vs. LOS until death				
Home	83 (50.3%)	38 (45.8%)		18.6
Group home	10 (6.1%)	7 (70%)		10.9
Nursing facility	72 (43.6%)	58 (80.6%)		12.6

*LOS *length of STAY

### Sputum culture findings

**Fig. 1:**
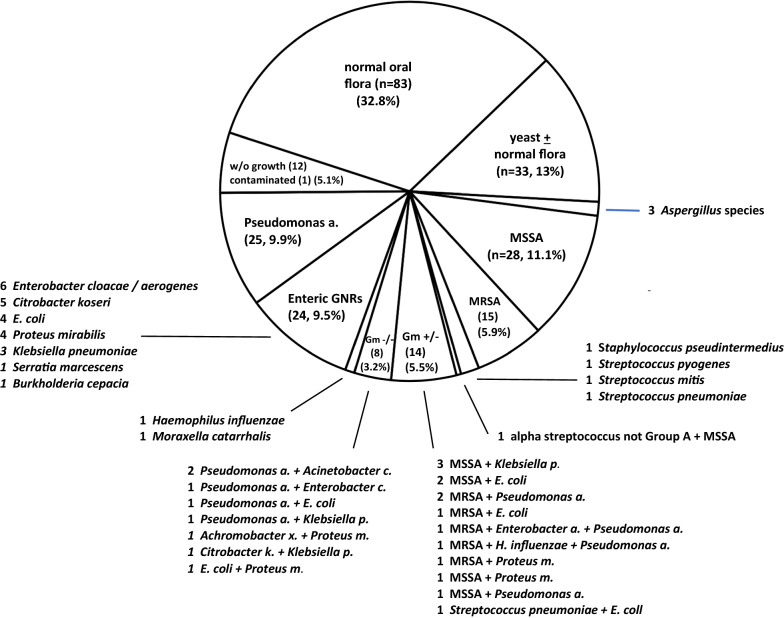
Distribution of all 253 sputum culture results from 165 patients with COVID-19 requiring intubation, March 1 to May 1

Results of 253 sputum cultures from 165 individual patients are shown in Fig. 1. Approximately one-third of cultures grew only normal oral flora (n = 83, 32.8%) and an additional group grew only yeast or normal flora plus yeast (n = 33, 13.0%). Three cultures grew *Aspergillus* species not felt to represent infection upon review of patient records and treatment regimens, while 12 (4.7%) were “without growth”. Influenza A/B and respiratory syncytial virus swabs were done in 21 patients and all were negative. Viral respiratory panels were done in 5 patients with one positive for coronavirus OC43. While not definitive, we posit based on chart review that most of these culture findings were not suggestive of bacterial infection.

A single Gram-positive bacterium was recovered from an additional 47 (18.6%) sputum cultures; these were predominantly *Staphylococcus aureus*, methicillin-susceptible (MSSA) (28, 11.1%) and methicillin-resistant (MRSA) (15, 5.9%). MRSA probes were done in 84 instances; six tests were positive but only three correlated with isolation of MRSA from sputum culture. Growth of a single Gram-negative bacterium constituted 51 cultures (20.2%) with *Pseudomonas aeruginosa* being most prevalent (n = 25, 9.9%). Cultures growing two or more bacterial species were noted in 23 instances (9.1%), predominantly combinations of Gram-positive and Gram-negative organisms (14 cases) and Gram-negative flora only (8 cases) with one culture having only Gram-positive flora. *Staphylococcus aureus* and *Pseudomonas aeruginosa* were the most common bacteria found in mixed cultures.

### Significance of sputum culture timing related to pneumonia diagnosis and intubation

In Table 2, sputum results are correlated with timing of cultures relative to hospital admission and intubation and likely clinical significance. Submission of sputum cultures was associated with signs and symptoms of infection, including fever, cough, and leukocytosis, and a radiographic study showing a new infiltrate or worsening of prior findings. Tracheal aspirate cultures were commonly ordered at the time of intubation. Sputum was not obtained due to lack of patient production of sputum or, in a minority of cases, death or change to palliative care prior to obtaining sputum adequate for culture. Overall, there were 114 expectorated sputa, 98 tracheal aspirates, and 110 labelled only as “sputum” (most obtained during periods of intubation, therefore likely tracheal aspirates). Seven bronchial lavage procedures were performed, with cultures yielding two *Pseudomonas a.,* one MSSA, and one yeast. Two transtracheal aspirations were performed and grew MRSA and yeast in one each. Six pleural fluid cultures were performed with one growing *Pseudomonas a.* matching a sputum isolate.

**Table 2:** Sputum culture results related to timing of pneumonia diagnosis and intubation

	PNA on ADM before intubation (n = 33)	PNA w/in 24 h of intubation (n = 165)	Late PNA ≥ 1 week after intubation (n = 80)	Colonization ≥ 1 week after intubation (n = 31)
No. (%) receiving antibiotics	(n = 18, 54.5%)	(n = 144, 87.3%)	(n = 80, 100%)	(n = 20, 64.5%)
No sputum obtained (50)*	26	21	3	–
Ordered, not done (6)	1	4	1	-
Without growth (12)	–	11	–	1
Normal oral flora only (83)	4	62	13	4
Contaminated (1)	1	–	–	–
Yeast ± normal oral flora (33)	–	16	12	5
Solitary Gram-positive (47)				
MSSA	–	17	9	2
MRSA	1	6	8	–
* Staph pseudintermedius*	–	–	1	–
* Streptococcus mitis*	–	1	–	–
* Streptococcus pyogenes*	–	1	–	–
* Streptococcus pneumoniae*	–	–	1	–
Solitary Gram-negative (51)				
* Pseudomonas aeruginosa*	–	3	14	8
* Escherichia coli*	–	4	–	–
* Proteus mirabilis*	–	1	2	1
* Citrobacter koseri*	–	2	1	2
* Enterobacter a/c*	–	1	3	2
* Klebsiella pneumoniae*	–	–	2	1
* Serratia marscescens*	–	–	1	–
* Burkholderia cepacia*	–	–	1	–
* Haemophilus influenzae*	–	1	–	–
* Moraxella catarrhalis*	–	1	–	–
Combination (Gram pos/pos) (1)				
Alpha strep not gr A, MSSA	–	–	–	1
Combination (Gram pos/neg) (14)				
MSSA, *E. coli*	–	2	–	–
MSSA, *Klebsiella p*	–	1	1	1
MSSA, *Proteus m*	–	–	–	1
MSSA, *Ps. a*	–	–	1	–
MRSA, *E. coli*	–	1	–	–
MRSA, *Enterobacter a., Ps. a*	–	1	–	–
MRSA, H*. influenzae, Ps. a*	–	1	–	–
MRSA, *Proteus m*	–	–	–	1
MRSA, *Ps. a*	–	–	2	–
* Strep pneumoniae, E. coli*	–	1	–	–
Combination (Gram neg/neg) (8)				
* Ps. a., Acinetobacter c*	–	1	–	1
* Ps. a., Enterobacter c*	–	–	1	–
* Ps. a., E. coli*	–	1	–	–
* Ps. a., Klebsiella p*	–	–	1	–
* Achromobacter x., Proteus m*	–	1	–	–
* Citrobacter k., Klebsiella p*	–	–	1	–
* E. coli, Proteus m*	–	–	1	–
Other fungi (3)				
Aspergillus species	–	3	–	–
Residence vs. culture results				
Home	20 (60.6% total)	84 (50.9%)	57 (71.2%)	21 (67.7%)
Group Home	3 (9.1%)	10 (6.1%)	4 (5.0%)	0 (0%)
Nursing Home	10 (30.3%)	71 (43.0%)	19 (23.8%)	10 (32.3%)
ADM CXR, not intubated (33)				
GGO only	30 (3 normal flora, yeast; 26 no sputum; 1 contaminated)			
Includes consolidation	3 (1 normal flora, 1 ordered, not done, 1 MRSA)			
CXR at intubation (165)				
Predom. interstitial, GGO	35 (22 normal flora, yeast; 6 no sputum, 4 no culture growth, 1 MSSA, 2 MRSA)			
Predom. consolidation	130 (56 normal flora, yeast; 15 no sputum; 7 no growth; 4 ordered, not done; 48 potential pathogens most common = 16 MSSA, 4 MRSA, 4 E. coli, 3 Ps. a.)			

Intubation culture vs. residence, outcome	Isolate nos	From group or nursing home no. (%)	Overall mortality (%)
Normal flora or yeast	78	44 (43.6%)	(56.4%)
MSSA only	17	12 (70.6%)	(64.7%)
MRSA only	6	6 (100.0%)	(100.0%)
*Pseudomonas a.* only	3	2 (66.7%)	(66.7%)
*Aspergillus* only	3	2 (66.7%)	(66.7%)
Any MSSA in culture	20	12 (60.0%)	(70.0%)
Any MRSA in culture	9	9 (100.0%)	(88.9%)
Any Ps. a. in culture	7	6 (85.7%)	(71.4%)

*PNA* pneumonia, *ADM* admission, *MSSA* methicillin-susceptible *Staphylococcus aureus*, *MRSA* methicillin-resistant *Staphylococcus aureus*, *Staph pseudintermedius* *Staphylococcus pseudintermedius*, *Enterobacter a./c*. *Enterobacter aerogenes/cloacae*, *Alpha strep not group A* alpha streptococcus not group A, *Ps. a.* *Pseudomonas aeruginosa, H. influenzae* *Haemophilus influenzae*, *Proteus m.* *Proteus mirabilis*, *E. coli* *Escherichia coli*, *Klebsiella p.* *Klebsiella pneumoniae*, *Acinetobacter c.* *Acinetobacter calcoaceticus*, *Achromobacer x*. *Achromobacter xylosoxidans*, *Citrobacter k.* *Citrobacter koseri*

### Admission radiographic and culture findings

Thirty-three patients had chest X-rays (CXRs) showing pneumonia upon admission but did not require intubation until four or more days later (range = 4–11 days) (Table 2). The majority were not producing sputum (26 = 78.8%) while 4 (12.1%) sputum cultures yielded normal flora only. One culture grew MRSA, one was ordered but not completed, and one was deemed to be contaminated. Admission CXRs for patients not requiring intubation initially revealed 30 (90.9%) had only interstitial “ground glass” opacities while the remaining 3 (9.1%) included alveolar airspace disease. The MRSA isolate was from a nursing home resident, associated with alveolar airspace disease, and judged clinically relevant and treated. Notably, 18 (54.5%) of these patients received intravenous antibacterials in addition to hydroxychloroquine and/or azithromycin.

### Observations at time of intubation

All 165 patients studied were known to be COVID-19 (+) and were in varying degrees of respiratory distress with worsening respiratory failure upon admission. Contemporaneous records note that all had or were evolving severe pneumonia within 24 h of intubation. No sputum was cultured in 25 (15.2%) cases, while there was no growth in 11 (6.7%), normal oral flora only in 62 (37.6%), and yeast ± normal oral flora in 16 (9.7%). Thus 114 (69.1% of study patients) did not have culture data directly supporting a diagnosis of bacterial infection at intubation. However, the remaining 51 (30.9% of study patients) did grow a potential pathogen, primarily bacterial (48 = 94.1% of pathogens) and a few *Aspergillus* species isolates. (3 = 5.9% of pathogens). *Staphylococcus aureus* was the most common solitary bacterial isolate upon intubation with methicillin-susceptible strains (17 = 33.3% of pathogens), nearly thrice the number of MRSA isolates (6 = 11.8% of pathogens). *Escherichia coli* (4 = 7.8%) and *Pseudomonas aeruginosa* (3 = 5.9%) were the most common of a variety of solitary gram-negative potential pathogens. Polymicrobial growth was seen in 10 cultures (19.6% of those with potential pathogens); MSSA, MRSA, and *Pseudomonas aeruginosa* were most commonly represented.

### Results of cultures from nonrespiratory sites

Numerous blood cultures were obtained throughout the study period; 40 were positive with 16 growing coagulase-negative staphylococcus. Five cultures from one patient grew *Pseudomonas a.* which was consistent with lung cultures. Two cultures grew *Streptococcus mitis* in the same patient, one grew *Streptococcus pneumoniae,* and one grew *Candida a.*; these were consistent with concurrent sputum culture results. The remaining blood cultures grew an additional *Pseudomonas a.* (1), *E. coli* (6), *Proteus m.* (3), *Streptococcus salivarius* (2), *Bifidobacterium* (2), *Bacillus sp.* (1) without correlation with sputum culture results. Twenty-six urine cultures were obtained within 7 days before or after one of the episodes of potential pneumonia studied. The isolates were *E. coli* (9), *Klebsiella p.* (5), *Pseudomonas a.* (2), *Enterococcus faecalis* (2), *Enterobacter sp.* (1), *Citrobacter* (1), *Aerococcus* (1), mixed gram-negative bacilli (4), and *Candida a.* (1). Only one isolate, an *E. coli*, matched the sputum of the same patient.

### Radiologic findings at time of intubation

Review of 2 to 4 CXRs and/or chest CT scans for each patient performed around intubation revealed 35 patients with interstitial or ground glass infiltrates to a much greater degree than alveolar disease and lobar infiltrates; a viral aetiology was generally suspected. Somewhat surprisingly there was little overlap between this group and the 33 patients not requiring intubation until 4 or more days following admission; seven patients were in both groups. Sputum specimens associated with this radiographic pattern yielded 2 MRSA and 1 MSSA (totalling 8.6% of results) versus 22 normal flora or yeast and 10 either without growth or without sufficient sputum for culture. The remaining 130 patients had a predominantly alveolar/lobar imaging pattern. Of these, 48 yielded bacterial isolates (37% of results) including 16 MSSA, 4 MRSA, 4 *E. coli*, and 3 *Pseudomonas a*. with the remaining isolates demonstrative of 56 normal flora/yeast, 7 no growth, and 15 no sputum obtained. Lengths of stay and mortality rates were very similar for the two groups despite differences in predominant radiographic pattern.

### Late pneumonias after intubation and sputum colonisation

Given extended hospitalisations (mean (SD) LOS of all patients = 19.9 days (14.4), range 2–90 days) and intubation and other invasive procedures, situations suggestive of new infection were not uncommon. Table 2 shows 80 episodes possibly consistent with late pneumonia after intubation; most patients only had one episode, but 7 patients had two, 4 patients had three, and 1 patient had five. In 29 instances (36.3% of those suggestive of late pneumonia), culture results were 4 (5% of total) no sputum obtained or sent, 13 (16.3% of total) normal oral flora only, and 12 (15% of total) yeast ± normal flora. With regard to potential pathogens isolated, the remaining 51 (63.8% of late pneumonias) cultures included 17 (33.3% of positives) with *Staphylococcus aureus,* now more equally divided between MSSA and MRSA (9 vs. 8). *Pseudomonas aeruginosa* (n = 14, 27.5% of positives) was the leading solitary gram-negative potential pathogen in late pneumonia.

Cultures judged to have sputum colonizers were found in 31 instances after intubation; these had a similar distribution of normal and potentially pathogenic microbial flora with *Pseudomonas* species most commonly recovered as shown in Table 2.

### Development of antimicrobial resistance (AMR) during hospitalisation

Notably, in 12 patients (11 with late pneumonias and 1 colonization) increasing antimicrobial resistance developed in bacteria during the course of the hospitalisations; see Table 3. Pneumonia organisms which developed AMR most commonly were 7 *Pseudomonas aeruginosa* with previously susceptible organisms acquiring resistance to many 3rd generation cephalosporins, broad-spectrum beta-lactam/beta-lactamase inhibitors, and sometimes carbapenems. Two *Staphylococcus aureus* transitioned from MSSA to MRSA. An *Enterobacter cloacae* developed new resistance to 3rd gen cephalosporin, ticarcillin/clavulanate, and ertapenem and new intermediate susceptibility to piperacillin/tazobactam and meropenem over 17 days after prior courses of carbapenem and an intervening course of piperacillin/tazobactam. An *Enterobacter aerogenes* developed new resistance to 3rd gen. cephalosporin, ciprofloxacin, and piperacillin/tazobactam over 8 days during courses of piperacillin/tazobactam followed by cefepime. One *Klebsiella* isolate acquired tetracycline resistance but was judged to be a colonizer. Of the 11 late pneumonia patients, only four (36.4%) had been on antibacterial agents prior to hospital admission but all of the pneumonia patients and the colonization patient in this group had received antibacterials around the time of Intubation. In each case of cultures consistent with late pneumonia, when susceptibility data became available antibiotics were modified to cover the new resistance patterns.

**Table 3:** Timing of antimicrobial resistance development during hospitalisation

Pt age, sex	Admit from	Med risk factors	Prior hosp^a^antibx^b^	Organism	Resistance acquired	Episode type and number^c^	Days post-adm/intubation
49 M	Home	DM	No, No	Ps. a	Aztreonam	Late PNA	37/28
					Ceftazidime	third	
					pip/tazo		
60 F	Home	DM	No, No	Ps. a	Imipenem	Late PNA	39/38
		fmr smoker				second	
73 F	Home	CVA	No, Yes	Ps. a	Aztreonam	Late PNA	23/20
		endomet CA	(amox/clav)		ticar/clav	second	
					meropenem		
75 M	NH	asthma	Yes, No	MSSA	Replaced by MRSA	Late PNA	12/12
		dementia				first	
		aspiration					
		EtOH abuse					
74 M	Home	OSA	No, No	Ps. a	ticar/clav	Late PNA	45/40
		AVMs				first	
74 F	Home	fmr smoker	No, Yes	Ps. a	Ceftazidime	Late PNA	11/10
			(oseltam)		then carba	first	
54 M	Home	DM	No, Yes	MSSA	Replaced by MRSA	Late PNA	23/20
		fmr smoker	(azithro)			second	
66 F	Home	OSA	No, No	Ps. a	ticar/clav	Late PNA	30/27
		seizures			then aztreo	first	
		inter lung dis			pip/tazo		
60 M	Home	DM	No, No	Enter a	Aztreonam	Late PNA	58/54
		fmr smoker			3 gen ceph	fourth	
		prostate CA			ticar/clav		
					ertapenem		
84 F	Home	afib	Yes, Yes	Ps. a	Aztreonam	Late PNA	30/30
		CVA	(doxy,		ceftazidime	second	
			amox/clav)		pip/tazo		
					meropenem		
72 M	Home	ESRD/HD	Yes, Yes	Enter c	3 gen ceph	Late PNA	20/11
		fmr smoker	(cefazolin)		pip/tazo	first	
		afib					
84 F	Home	DM	No, No	Klebs p	tcn	Colonization	26/26
		hx DVT/PE				second	

^a^Prior hospitalization within 90 days of admission

^b^Antibiotics within 30 days of admission to hospital

^c^Episode defined as positive sputum culture, occurring after intubation, categorized as either late pneumonia or colonization and numbered consecutively

*NH* nursing home, *DM* diabetes mellitus, *fmr smoker* former smoker, *endomet CA* endometrial cancer, *EtOH abuse* alcohol abuse, *OSA* obstructive sleep apnea, *AVMs* arteriovenous malformations, *inter lung disease* interstitial lung disease, *prostate CA* prostate cancer, *CVA* cerebrovascular accident, *ESRD/HD* endstage renal disease/haemodialysis, *afib* atrial fibrillation, *hx DVT/PE* history of deep venous thrombosis/pulmonary embolism, *amox/clav* amoxicillin/clavulanate, *oseltam* oseltamivir, *azithro* azithromycin, *doxy* doxycycline, *Ps. a.* Pseudomonas aeruginosa, *MSSA* methicillin-susceptible *Staphylococcus aureus*, *Enter a*. Enterobacter aerogenes, *Enter c*. *Enterobacter cloacae*, *Klebs p.* *Klebsiella pneumoniae*, *pip/tazo* piperacillin/tazobactam, *ticar/clav* ticarcillin/clavulanate, *carba* carbapenem, *aztreo* aztreonam, *3 gen ceph* third generation cephalosporin, *tcn* tetracycline, *PNA* pneumonia

## Discussion

We reviewed critically ill COVID-19 inpatients with respiratory failure requiring intubation. Mortality rate was 62.4% (95% CI: 54.6%, 69.8%) reflecting many patients' chronic health problems and advanced age. These outcomes were similar to other reports from the United States [35]. Nursing home residency was associated with the highest mortality; younger patients from group homes also had prolonged hospital stays and significant mortality. In most patients not requiring intubation within three days of admission, lack of sputum production or growth of normal oral flora and variations was associated with a ground glass appearance of CXR infiltrates suggesting viral pneumonia.

Around intubation, a sizeable fraction of cultures (69.1%) were still not strongly supportive of bacterial infection and many of these cultures were from 35 of the 165 patients with radiologic imaging showing predominance of interstitial infiltrates and ground glass opacities over dense lobar airspace disease. However, many cultures around intubation also grew solitary to mixed cultures of potential bacterial respiratory pathogens. *Staphylococcus aureus*, predominantly MRSA, *Pseudomonas aeruginosa*, and various enteric Gram-negative bacteria were also recovered, a pattern also seen elsewhere [38]. MRSA recovery was associated with group and nursing home residency and the highest overall mortality rate compared with other sputum isolates. Beyond one week following intubation, both new episodes of suspected pneumonia and instances of colonization had the same spectrum of culture results. However, methicillin-resistance became more common among *Staphylococcus aureus* and *Pseudomonas aeruginosa* also was more common which could reflect early treatment with a non-antipseudomonal beta-lactam such as ceftriaxone ± azithromycin as well as the ICU environment. Significantly, 12 cultures (10.8%) out of 111 beyond one week of intubation grew organisms, predominantly *Pseudomonas aeruginosa,* that had developed increased antimicrobial resistance. Contrary to expectations, most worsening of AMR was in patients from home rather than nursing home residents. This probably reflects the earlier and higher mortality of individuals from nursing homes. These individuals were more likely to grow *Staphylococcus aureus* and/or *Pseudomonas aeruginosa* from sputum at the time of intubation but there were relatively few longterm survivors. Development of AMR occurred late (average = 29.5 days, range = 11–58 days) after admission likely explaining the difference of our findings from studies that followed hospitalised patients for much shorter periods of time. Most episodes (54.5–100% depending on type of suspected pneumonia) in our study patients were treated with 5 days or more of broad spectrum antibiotics and even the episodes most consistent with sputum colonization were treated with antibiotics 64.5% of the time contributing to risk of AMR.

Our findings show a higher percentage of patients with bacterial and fungal growth in sputum cultures than some reports [16, 38]. Our patients were sicker than in a study of hospitalised but not intubated patients and there was longer patient follow up [31]. Microorganisms recovered in our study were similar in some regards to published data [31] but with relatively few *Streptococcus pneumoniae, Haemophilus*, and *Moraxella* isolates compared with other studies [28, 33, 39]. These common community-acquired pneumonia pathogens could have been suppressed by azithromycin or other antibiotics given prior to or during the hospitalizations studied. Testing was not done for *Mycoplasma pneumoniae* which was a common superinfecting pathogen in one review [40]. Also, the three *Aspergillus* isolates did not represent invasive disease which was a concern of several published reviews and reports [40–43]. We only found one viral co-infection (coronavirus OC43) based on very few tests; some reports have shown very low viral coinfection rates [33] while others have found rates up to 8% [44] in COVID-19. Our study does suggest that following admission and intubation, COVID-19 pneumonia patients have the same predisposition to colonization and infections due to nosocomial pathogens well-known in hospital-acquired and ventilator-associated pneumonias. The literature on bacterial sputum cultures in COVID-19 report high rates and duration of antibiotic use [16, 17, 45–48]. We observed this as well and demonstrated antimicrobial resistance development in over 10% of bacterial isolates late in patients' hospitalisations; this had been predicted [47] but also has been a topic of considerable debate [49–54].

Since completion of our data analysis in August 2020, other ICU COVID-19 studies have been published showing similar microbial flora recovered (e.g., *Staphylococcus aureus*, *Pseudomonas a*., *Acinetobacter* spp., *Klebsiella* spp.) [55–57], late infections after intubation [55], and recovery of multidrug-resistant pathogens [58]. These also extend results to a wider geographic area including South America [56] and the Middle East [59], as well as patient populations such as cancer patients [60] and postmortem studies [57]. An Italian paper showed bronchoalverolar lavage from 24 critically ill COVID-19 patients tended to grow gram-negative bacteria "predisposed to multidrug resistance" while 24 matched non-COVID-19 controls had more commensal flora such as *Haemophilus* sp. and streptococci [61]. Another reported 7 relatively young COVID-19 ICU patients with few comorbidities except obesity harbored carbapenemase-producing *Klebsiella pneumoniae* [62]. There was rapid spread of MDR-*Escherichia coli* in a cluster of 4 COVID-19 patients in Maryland, USA, probably related to facility crowding and overburdened staff [63].

Similar to our findings, some areas have seen low rates of COVID-19-associated fungal infections [64] while multiple fungal types were prevalent in Egypt [59]. Candidiasis in COVID-19 cases has been associated with the ICU setting, central vascular access, and broad spectrum antibiotic use [64]. A German prospective study on invasive pulmonary aspergillosis in critically ill COVID-19 patients suggested a four-fold higher rate (34%) in 32 COVID-19 patients compared with 8% of matched controls [65]. Mucormycosis was a significant fungal infection in Egypt [59] and notably India [66] though more often rhinoorbital or rhinocerebral than pulmonary in the latter.

A review of 49 published references on secondary pulmonary infections complicating COVID-19 pneumonia during 2020 gave an average incidence of 16% for bacterial causes and 6.4% for fungal etiologies though ranges were quite broad [67]. Cases of proven healthcare associated pneumonia may be underdiagnosed as fewer bronchoscopies are performed to avoid risks to staff [68]. It is clear that bacterial and fungal coinfections and superinfections of COVID-19 is an evolving topic. Influenza has been shown to have specific virulence factors that predispose to certain pathogens such as *Streptococcus pneumoniae* [8] and possibly *Aspergillus* [69]. In contrast, COVID-19 superinfections may be more related to crowded healthcare settings, immunosuppressive therapies, and invasive procedures [8]. The timing and spectrum of pathogens differs due to many factors. Patient treatment with teichoplanin, which has potent gram-positive activity, increased infections with *Pseudomonas* and other gram-negative infections relative to staphylococci [70]. Early versus late superinfections differ in character [71] with longer durations of hospital stay and mechanical ventilation increasing infection rates [72, 73]. Also, the types of infections encountered may be changing due to newer approaches to therapy (positioning, corticosteroids, antibiotic selection, antiviral drugs). Rapid testing with a multiplex PCR assay for pathogen/resistance diagnosis can reduce antibiotic use and contain AMR [74] but potentially with increased cost. In resource limited settings, decreased availability of diagnostic tests and access to expensive antimicrobials may make AMR a critical issue [75]. Future prospective trials with more rigorous diagnostic studies and comparing empiric antibacterial and antifungal use with reduced prescribing is indicated [8]. Rigorous antibiotic stewardship approaches to COVID-19 management offer the opportunity to control antimicrobial use and decrease AMR [76, 77].

Strengths of our study include large size and extended follow up. This data from the United States allow further comparison with Asia and Europe. Our health system is not a large tertiary referral center, so our experience may be in line with similar facilities in suburban areas of the US which are becoming pandemic epicenters. Our mix of patients ranging from “healthy” individuals coming from home to older, debilitated individuals residing in nursing facilities may also guide management based on populations served.

Limitations of our study include lack of use of diagnostic tools targeting atypical bacterial infections, e.g. mycoplasma, and the lack of procalcitonin values. Influenza and respiratory syncytial virus studies were done in a minority of patients as infections with these pathogens had waned in our area by the spring. Only some patients received tocilizumab which could influence mortality data. Hydroxychloroquine and azithromycin are no long being given routinely to COVID-19 patients; remdesivir was not available during the study time frame and dexamethasone was not yet considered standard of care.

## Conclusions

Based on our findings in severe COVID-19 pneumonia, we recommend: (1) empiric antibacterials should be used sparingly in patients presenting without sputum production and with a radiographic ground glass interstitial pattern suggestive of viral aetiology; (2) consider discontinuation of empiric antibiotics after 48 h in patients without sputum to culture despite adequate access or who have no growth or “normal flora/yeast”; however, this must be balanced against the risk of bacterial pathogen suppression by prior or current antimicrobial regimens and also take into account other clinical findings, results of radiographic studies, and the overall trend in clinical progress; (3) with longer duration of hospitalisation, sputum cultures increasingly reflect hospital-acquired microbial flora so length of stay and “clinical trajectory” are critical in deciding to use antibiotics and selection of agents; (4) culture results, antibiotic use, and clinical outcomes in COVID-19 patients should be reviewed periodically with changes guided by principles of antimicrobial stewardship.

## Data Availability

The datasets generated and/or analysed during the current study are not publicly available per the MLHS Institutional Review Board due to the chance they may contain identifiable protected health information but are available from the corresponding author on reasonable request such as for editorial review.

## Abbreviations

AMR: Antimicrobial resistance
CI: Confidence interval
CXR: Chest X-Ray
Enteric GNRs: Enteric gram-negative rod bacteria
ICU: Intensive care unit
LOS: Length of stay
MERS: Middle eastern respiratory syndrome
MLHS: Main Line Health System
MRSA: Methicillin-resistant *Staphylococcus aureus*
MSSA: Methicillin-susceptible *Staphylococcus aureus*
PCR: Polymerase chain reaction
SARS: Severe acute respiratory syndrome
SD: Standard deviation

## References

[1] Kumar A, Arora A, Sharma P (2020). Clinical Features of COVID-19 and factors associated with severe clinical course: a systematic review and meta-analysis. SSRN [Preprint]..

[2] Chua RL, Lukassen S, Trump S (2020). COVID-19 severity correlates with airway epithelium-immune cell interactions identified by single-cell analysis. Nat Biotechnol.

[3] Hu B, Huang S, Yin L (2021). The cytokine storm and COVID-19. J Med Virol.

[4] Zhou F, Yu T, Du R (2020). Clinical course and risk factors for mortality of adult inpatients with COVID-19 in Wuhan, China: a retrospective cohort study. Lancet.

[5] Varga Z, Flammer AJ, Steiger P (2020). Endothelial cell infection and endotheliitis in COVID-19. Lancet.

[6] Bradley BT, Maioli H, Johnston R (2020). Histopathology and ultrastructural findings of fatal COVID-19 infections in Washington State: a case series. The Lancet.

[7] Chen X, Liao B, Cheng L (2020). The microbial coinfection in COVID-19. Appl Microbiol Biotechnol.

[8] Rawson TM, Wilson RC, Holmes A (2021). Understanding the role of bacterial and fungal infection in COVID-19. Clin Microbiol Infect.

[9] MacIntyre CR, Chughtai AA, Barnes M (2018). The role of pneumonia and secondary bacterial infection in fatal and serious outcomes of pandemic influenza a(H1N). BMC Infect Dis.

[10] Abelenda-Alonso G, Rombauts A, Gudiol C (2020). Influenza and bacterial coinfection in adults with community-acquired pneumonia admitted to conventional wards: risk factors, clinical features, and outcomes. Open Forum Infect Dis.

[11] Waldeck F, Boroli F, Suh N (2020). Influenza-associated aspergillosis in critically-ill patients—a retrospective bicentric cohort study. Eur J Clin Microbiol Infect Dis.

[12] Yap FH, Gomersall CD, Fung KS (2004). Increase in methicillin-resistant* Staphylococcus aureus* acquisition rate and change in pathogen pattern associated with an outbreak of severe acute respiratory syndrome. Clin Infect Dis.

[13] Memish ZA, Almasri M, Turkestani A, Al-Shangiti AM, Yezli S (2014). Etiology of severe community-acquired pneumonia during the 2013 Hajj-part of the MERS-CoV surveillance program. Int J Infect Dis.

[14] World Health Organization. COVID-19 Clinical Management. Living Guidance 25 January 2021. Available from: https://www.who/int/iris/handle/10665/338882. Accessed 6 Mar 2021.

[15] Rawson TM, Moore LSP, Zhu N et al. Bacterial and fungal co-infection in individuals with coronavirus: a rapid review to support COVID-19 antimicrobial prescribing [Preprint]. Clin Infect Dis. 2020;530. 10.1093/cid/ciaa530. 10.1093/cid/ciaa530 PMC7197596 32358954

[16] Karami Z, Knoop BT, Dofferhoff ASM (2021). Few bacterial co-infections but frequent empiric antibiotic use in the early phase of hospitalized patients with COVID-19: results from a multicentre retrospective cohort study in The Netherlands. Infect Dis (Lond).

[17] Grau S, Echeverria-Esnal D, Gómez-Zorrilla S (2021). Evolution of antimicrobial consumption during the first wave of COVID-19 pandemic. Antibiotics.

[18] Vaughn VM, Gandhi T, Petty LA (2020). Empiric antibacterial therapy and community-onset bacterial co-infection in patients hospitalized with COVID-19: a multi-hospital cohort study. Clin Infect Dis.

[19] Somers EC, Eschenauer GA, Troost JP (2020). Tocilizumab for treatment of mechanically ventilated patients with COVID-19. Clin Infect Dis.

[20] Horby P, Lim WS, Emberson JR, RECOVERY Collaborative Group (2021). Dexamethasone in hospitalized patients with COVID-19. N Engl J Med.

[21] Dimmig LM, Wu D, Gold M (2020). IL6 inhibition in critically ill COVID-19 patients is associated with increased secondary infections. medRxiv.

[22] Antimicrobial resistance in the age of COVID-19 [Editorial]. Nat Microbiol. 5:779 (2020). 10.1038/s41564-020-0739-4. 10.1038/s41564-020-0739-4 32433531

[23] Getahun H, Smith I, Trivedi K (2020). Tackling antimicrobial resistance in the COVID-19 pandemic. Bull World Health Organ.

[24] Rodríguez-Baño J, Rossolini GM, Schultsz C (2021). AMR research in a post-pandemic world: Insights on antimicrobial resistance research in the COVID-19 pandemic. J Global Antimicrob Resist.

[25] Rossato L, Negrão FJ, Simionatto S (2020). Could the COVID-19 pandemic aggravate antimicrobial resistance?. Am J Infect Control.

[26] Adler H, Ball R, Fisher M (2020). Low rate of bacterial co-infection in patients with COVID-19. The Lancet Microbe.

[27] Youngs J, Wyncoll D, Hopkins P (2020). Improving antibiotic stewardship in COVID-19: bacterial co-infection is less common than with influenza. J Infect.

[28] Zhu X, Ge Y, Wu T (2020). Co-infection with respiratory pathogens among COVID-2019 cases. Virus Res.

[29] Huttner BD, Catho G, Pano-Pardo JR (2020). COVID-19: don’t neglect antimicrobial stewardship principles!. Clin Microbiol Infect.

[30] Verroken A, Scohy A, Gerard L (2020). Co-infections in COVID-19 critically ill and antibiotic management: a prospective cohort analysis. Crit Care.

[31] Hughes S, Troise O, Donaldson H (2020). Bacterial and fungal coinfection among hospitalised patients with COVID-19: a retrospective cohort study in a UK secondary care setting. Clin Microbiol Infect.

[32] Garcia-Vidal C, Sanjuan G, Moreno-García E (2021). Incidence of co-infections and superinfections in hospitalized patients with COVID-19: a retrospective cohort study. Clin Microbiol Infect.

[33] Contou D, Claudinon A, Pajot O (2020). Bacterial and viral co-infections in patients with severe SARS-CoV-2 pneumonia admitted to a French ICU. Ann Intensive Care.

[34] Neto AGM, Lo KB, Wattoo A (2021). Bacterial infections and patterns of antibiotic use in patients with COVID-19. J Med Virol.

[35] Nori P, Cowman K, Chen V (2021). Bacterial and fungal coinfections in COVID-19 patients hospitalized during the New York City pandemic surge. Infect Control Hosp Epidemiol.

[36] Clancy CJ, Nguyen MH (2020). Coronavirus disease 2019, superinfections, and antimicrobial development: what can we expect?. Clin Infect Dis.

[37] M. Vaillancourt, Jorth P. The unrecognized threat of secondary bacterial infections with COVID-19. mBio. 2020;11. 10.1128/mBio.01806-20. 10.1128/mBio.01806-20 PMC7419722 32769090

[38] Dudoignon E, Caméléna F, Deniau B (2021). Bacterial pneumonia in COVID-19 critically ill patients: a case series. Clin Infect Dis.

[39] Adler H, Ball R, Fisher M (2020). Low rate of bacterial co-infection in patients with COVID-19. Lancet Microbe.

[40] Lansbury L, Lim B, Baskaran V, Lim WS (2020). Co-infections in people with COVID-19; a systemic review and meta-analysis. J Infection.

[41] Wu C-P, Adhi F, Highland K (2020). Recognition and management of respiratory coinfection and secondary pneumonia in patients with COVID-19. Cleveland Clin J Med.

[42] Falcone M, Tiseo G, Giordano C (2021). Predictors of hospital-acquired bacterial and fungal superinfections in COVID-19: a prospective observational study. J Antimicrob Chemother.

[43] Borman AM, Palmer MD, Fraser M (2020). COVID-19-associated invasive aspergillosis: data from the UK National Mycology Reference Laboratory. J Clin Microbiol.

[44] Kim KW, Deveson IW, Pang CNI (2021). Respiratory viral co-infections among SARS-CoV-2 cases confirmed by virome capture sequencing. Sci Rep.

[45] Zhou P, Liu Z, Chen Y (2020). Bacterial and fungal infections in COVID-19 patients: a matter of concern [letter to the editor]. Infect Control Hosp Epidemiol.

[46] Cheng B, Hu J, Zuo X (2020). Predictors of progression from moderate to severe coronavirus disease 2019: a retrospective cohort. Clin Micro Infect.

[47] Bengoechea JA, Bamford CG (2020). SARS-CoV-2, bacterial co-infections, and AMR: the deadly trio in COVID-19?. EMBO Mol Med.

[48] Townsend L, Hughes G, Kerr C, et al. Bacterial pneumonia coinfection and antimicrobial therapy duration in SARS-CoV-2 (COVID-19) infection. JAC-Antimicrob Resist. 2020;2. 10.1093/jacamr/dlaa071. 10.1093/jacamr/dlaa071 PMC7446659 32864608

[49] Vaillancourt M, Jorth P (2020). The unrecognized threat of secondary bacterial infections with COVID-19. MBio.

[50] Rawson TM, Moore LSP, Castro-Sanchez E (2020). COVID-19 and the potential long-term impact on antimicrobial resistance. J Antimicrob Chemother.

[51] Clancy CJ, Buehrle DJ, Nguyen MH (2020). PRO: the COVID-19 pandemic will result in increased antimicrobial resistance rates. JAC Antimicrob Resist..

[52] Callignon P, Beggs JJ (2020). CON: COVID-19 will not result in increased antimicrobial resistance prevalence. JAC Antimicrob Resist..

[53] Mirzaei R, Goodarzi P, Asadi M (2020). Bacterial co-infections with SARS-CoV-2. IUBMB Life.

[54] Intra J, Sarto C, Beck E (2020). Bacterial and fungal colonization of the respiratory tract in COVID-19 patients should not be neglected. Am J of Infect.

[55] Pickens CO, Gao CA, Cuttica MJ et al. Bacterial superinfection pneumonia in patients mechanically ventilated for COVID-19 pneumonia. Am J Resp Crit Care Med. 10.1164/rccm.202106-1354OC. 10.1164/rccm.202106-1354OC PMC8534629 34409924

[56] Cataño-Correa JC, Cardona-Arias JA, Porras Mancilla JP (2021). Bacterial superinfection in adults with COVID-19 hospitalized in two clinics in Medellín-Colombia, 2020. PLoS ONE.

[57] Clancy CJ, Schwartz IS, Kula B (2021). Bacterial superinfections among persons with coronavirus disease 2019: a comprehensive review of data from postmortem studies. Open Forum Infect Dis.

[58] Grasselli G, Scaravilli V, Mangioni D (2021). Hospital-acquired infections in critically ill patients with COVID-19. Chest.

[59] Meawed TE, Ahmed SM, Mowafy SMS (2021). Bacterial and fungal ventilator associated pneumonia in critically ill COVID-19 patients during the second wave. J Infect Public Health.

[60] Gudiol C, Durà-Miralles X, Aguilar-Company J (2021). Co-infections and superinfections complicating COVID-19 in cancer patients: a multicentre, international study. J Infect.

[61] Gaibani P, Viciani E, Bartoletti M (2021). The lower respiratory tract microbiome of critically ill patients with COVID-19. Sci Rep.

[62] Montrucchio G, Corcione S, Sales G (2020). Carbapenem-resistant Klebsiella pneumoniae in ICU-admitted COVID-19 patients: Keep an eye on the ball. J Glob Antimicrob Resist..

[63] Patel A, Emerick M, Cabunoc MK (2021). Rapid spread and control of multidrug-resistant gram-negative bacteria in COVID-19 patient care units. Emerg Infect Dis.

[64] Fekkar A, Lampros A, Mayaux J (2021). Occurrence of invasive pulmonary fungal infections in patients with severe COVID-19 admitted to the ICU. Am J Respir Crit Care Med.

[65] Lahmer T, Kriescher S, Herner A (2021). Invasive pulmonary aspergillosis in critically ill patients with severe COVID-19 pneumonia: results from the prospective AspCOVID-19 study. PLoS ONE.

[66] Al-Tawfiq JA, Alhumaid S, Alshukairi AN (2021). COVID-19 and mucormycosis superinfection: the perfect storm. Infection.

[67] Chong WH, Saha BK, Ananthakrishnan R (2021). State-of-the-art review of secondary pulmonary infections in patients with COVID-19 pneumonia. Infection.

[68] Wicky PH, Niedermann MS, Timsit JF (2021). Ventilator-associated pneumonia in the era of COVID-19 pandemic: how common and what is the impact?. Crit Care.

[69] Oliva A, Ceccarelli G, Borrazzo C (2021). Comparison of clinical features and outcomes in COVID-19 and influenza pneumonia patients requiring intensive care unit admission. Infection.

[70] Ceccarelli G, Alessandri F, Oliva A (2021). Superinfections in patients treated with Teicoplanin as anti-SARS-CoV-2 agent. Eur J Clin Invest.

[71] Bassetti M, Kollef MH, Timsit JF (2020). Bacterial and fungal superinfections in critically ill patients with COVID-19. Intensive Care Med.

[72] Musuuza JS, Watson L, Parmasad V (2021). Prevalence and outcomes of co-infection and superinfection with SARS-CoV-2 and other pathogens: a systematic review and meta-analysis. PLoS ONE.

[73] Buehler PK, Zinkernagel AS, Hofmaenner DA (2021). Bacterial pulmonary superinfections are associated with longer duration of ventilation in critically ill COVID-19 patients. Cell Rep Med.

[74] Maataoui N, Chemali L, Patrier J et al. Impact of rapid multiplex PCR on management of antibiotic therapy in COVID-19-positive patients hospitalized in intensive care unit. Eur J Clin Microbiol Infect Dis. 2021:1–8. 10.1007/s10096-021-04213-6. (Epub ahead of print. PMID: 33733394; PMCID: PMC7968559) 10.1007/s10096-021-04213-6 PMC7968559 33733394

[75] Lucien MAB, Canarie MF, Kilgore PE (2021). Antibiotics and antimicrobial resistance in the COVID-19 era: perspective from resource-limited settings. Int J Infect Dis.

[76] Para O, Caruso L, Ronchetti M (2021). Superinfection with difficult-to-treat bacteria in COVID-19 patients: a call for compliance with diagnostic and antimicrobial stewardship. Intern Emerg Med.

[77] Rawson TM, Ming D, Ahmad R (2020). Antimicrobial use, drug-resistant infections and COVID-19. Nat Rev Microbiol.

